# A Higher Professional Standard

**Published:** 1892-04

**Authors:** 


### Editorial Department.

F. E. DANIEL, M. D., EDITOR AND PUBLISHER.

THIS JOURNAL, by unanimous vote of the members, was made the Official Organ of the Austin District Medical Society.

COLLABORATORS:

*Dr. R. M. Swearingen, Austin.*
*Prof. B. E. Hadra, M. D., Galveston.*
*Prof. Geo. Cupples, M. D., San Antonio.*
*Dr. T. C. Osborn, Cleburne, Texas.*
*Dr E. J. Doering, Chicago.*
*Dr. E. J. Beall, Fort Worth.*
*Dr Odo Betz, Germany.*
*Prof. W. B. Rogers, M. D., Memphis.*
*Dr. J. W. McLaughlin, Austin.*
*Dr. T. J. Tyner, Austin.*
*Prof. J. F. Y. Paine, M. D., Galveston.*
*Dr. R. H. L. Bibb, Mexico.*
*Dr. T. J. Bennett. Austin.*
*Dr. Bat Smith, Wharton.*
*Dr. E. Meierhof, New York.*
*L. H. Luce, M. D., West Tisbury, Mass.*

### A HIGHER PROFESSIONAL STANDARD.

Closing a sensible article on this subject, the *St. Louis Clinique* says:

"We have heard a man insist upon a higher grade course of study for students and closer examinations for graduates, while it was an open secret that his own methods were questionable and his personal character rotten. Indeed, it is often such fellows who make most noise, and, as a consequence, excite the most disgust. Let the work of reformation which has begun in our schools be carried on in the profession until our ranks are free from those who disgrace themselves and all connected with them. There are enough of honest, right-minded men in the profession to freeze out or reform all these fellows—half doctors and half quacks. What we need is a recognition of a man's true standard, and the courage to treat him according to our convictions and his deserts."

The "recognition" is easy enough; the "need" is the courage, —the backbone, to make a summary example of the wolf in sheep's clothing who, robed with the dignity of membership in our associations, and wearing, perhaps, the mantle of official honor, stoops secretly to the devices of the quack.

The question of a higher professional standard is an important one, and one difficult of solution. In Texas we have well nigh despaired of it,—despaired of bringing all the profession up to the standard of a few shining examples.

It was hoped that through the JOURNAL the profession could in time be brought to represent in its State Association the highest element and best type of professional character; but we have failed of that assistance from the Association indispensably necessary to the success of our efforts.

The JOURNAL has exposed quackery in and out of the profession, and in one notable instance has brought to the attention of the Association a species of open and unblushing quackery on the part of a member high in favor, calculated to bring reproach upon the body medical, and to scandalize the Association; but they were lacking in the courage to relegate him to his proper sphere; and to-day the Association occupies the remarkable attitude of having pardoned in one, an offense for which (or a very similar one) they once before expelled three members *in absentia.*

How can a higher standard of professional character be attained? By requiring a preliminary examination for matriculation and a three or four years' graded course for graduation, the colleges can do much to raise the standard of education. But, in what way can physicians be made to observe the requirements of the code and lead correct professional lives, who are too weak to resist the temptations to resort to short cuts to make money, the tricks and devices of the charlatan or quack, or whose desire for gain is stronger than their moral sense or professional pride? It can only be done by the profession themselves, and if they have not the courage to do it when they have the opportunity, we had as well despair of reformation in that regard, as of reforming and making good citizens of all the criminally inclined.

But by promptly ostracising an offender against the code and morals, and thus making him take his proper place in the ranks of the quacks, where he properly belongs, a wholesome fear of consequences may deter others who are inclined to quackery, and make them behave themselves. When a physician who has hitherto deported himself with proper professional dignity stoops to the devices of the quack, or is tempted by the hope of gain to resort to unprofessional means, as in a well known instance referred to above, and another recently in Cincinnati; were the medical profession to at once discard and discountenance him, cease to affiliate with him, it would do much toward "elevating the standard of professional character."

It may sound paradoxical to speak of making a physician ethical; but it is professional pride of character alone which causes many a man to adhere to the code, just as it is pride and the fear of public censure that makes the soldier brave. Many a man of moral courage is physically a coward and *vice versa.* Few private soldiers would seek the bubble reputation at the cannon's mouth, or stand fire at all, were it not for pride and fear of consequences. For those of high moral courage in the medical profession no compulsion and no code is necessary; they are physicians because they are gentlemen, but we all know there are men in our ranks who are frequently guilty of unprofessional conduct, and when evidences of it come to the knowledge of the profession, especially in its organized state, no maudlin sentiment nor mistaken notion of charity, nor misplaced "respect for gray hairs;" no consideration of past good behavior should screen the offender. The higher in standing the further the fall; the more intelligent the offender the less excuse for his defection. He should be dealt with promptly, and made to realize that he is in the wrong pew, and that he cannot pose as a physician and practice as a quack.

Yes, there are "enough good men in the profession to rid it of these fellows;" but alas, they have not the "courage to treat these fellows according to their deserts." "White-washing" is one of the modern devices of organized medicine. Will the Cincinnati Medical College, for instance, and his Medical Society expel Dr. Amick, or will the latter, through its judicial council, "exonerate him from accusations of conduct unbecoming a physician," *a la* Waco? We shall see.

